# Supplementary material for: Hormone Receptor Expression Variations in Normal Breast Tissue: Preliminary Results of a Prospective Observational Study
Source: J Pers Med. 2021 May 8;11(5):387. doi: 10.3390/jpm11050387 (PMC8150273; doi:10.3390/jpm11050387)
Supplement: Supplementary file 1 [file jpm-11-00387-s001.zip › jpm-1167522-supplementary.pdf]

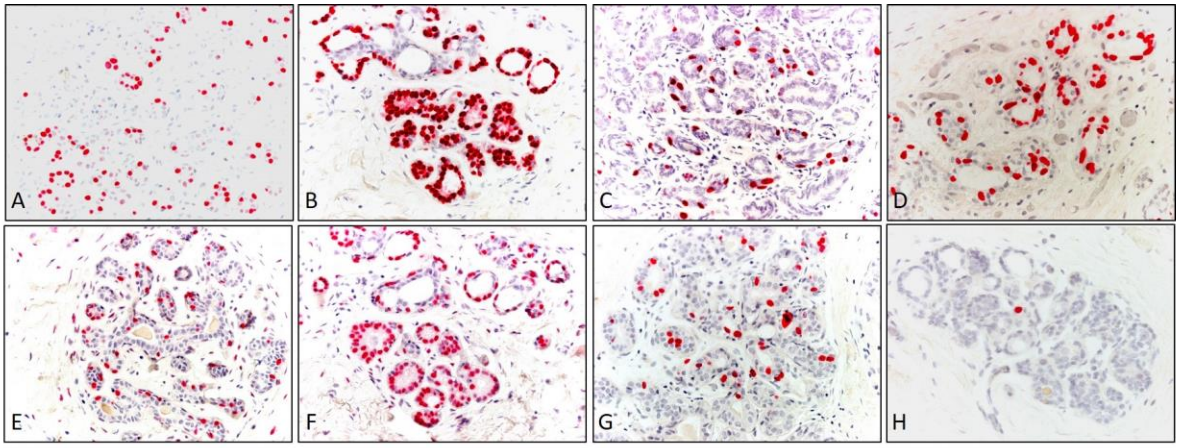

Figure S1: Examples of positivities obtained with ER, PR, AR and Ki67. ER expression in a young pre-menopausal woman **(A)** the number of positive nuclei is lower that that observed in a post-menopausal woman **(B)**. PR expression showed the opposite trend, being lower in pre-menopause **(C)** than in post-menopause **(D)**. AR expression showed a trend similar to ER, being higher in pre-menopause **(E)** than in post-menopause **(F)**. Ki67 stained some acinar nuclei in pre-menopause (G) while was almost absent in post-menopause (H).

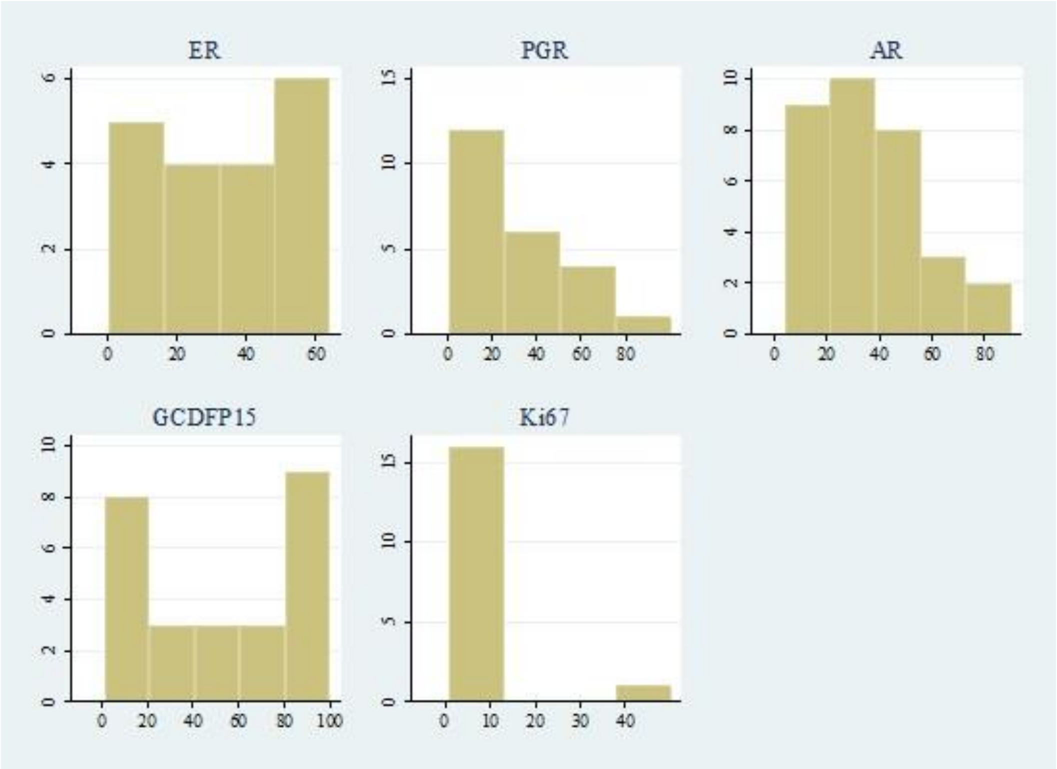

Figure S2: Histograms of markers used in the study.

Table S1: Results of the Shapiro-Wilk test of normality conducted on the markers used in the study.

Variable	Obs	W	p-value
ER	19	0.90849	0.069
PGR	23	0.91050	0.042
AR	32	0.95571	0.209
GCDFP15	26	0.93359	0.094
Ki67	17	0.48178	<0.001

For ER and Ki67 the hypothesis of normal distribution is rejected.
